# Neural subgroups in unaffected first-degree relatives of patients with bipolar disorder during emotion regulation

**DOI:** 10.1017/S0033291724003593

**Published:** 2025-02-12

**Authors:** Hanne Lie Kjærstad, Florien Ritsma, Klara Coello, Sharleny Stanislaus, Klaus Munkholm, Maria Faurholt-Jepsen, Julian Macoveanu, Anne Juul Bjertrup, Maj Vinberg, Lars Vedel Kessing, Kamilla Woznica Miskowiak

**Affiliations:** 1 Neurocognition and Emotion in Affective Disorders (NEAD) Centre, Psychiatric Centre Copenhagen, Mental Health Services, Capital Region of Denmark, and Department of Psychology, University of Copenhagen, Copenhagen, Denmark; 2 Copenhagen Affective Disorder research Centre (CADIC), Psychiatric Centre Copenhagen, Frederiksberg Hospital, Mental Health Services, Frederiksberg, Capital Region of Denmark; 3 Department of Clinical Medicine, Faculty of Health and Medical Sciences, University of Copenhagen, Copenhagen, Denmark; 4 The Early Multimodular Prevention and Intervention Research Institution (EMPIRI), Mental Health Centre, Northern Zealand, Copenhagen University Hospital – Mental Health Services CPH, Copenhagen, Denmark

**Keywords:** bipolar disorder, cluster analysis, emotion regulation, endophenotypes, fMRI, relatives

## Abstract

**Background:**

Impaired emotion regulation has been proposed as a putative endophenotype in bipolar disorder (BD). Functional magnetic resonance imaging (fMRI) studies investigating this in unaffected first-degree relatives (UR) have thus far yielded incongruent findings. Hence, the current paper examines neural subgroups among UR during emotion regulation.

**Methods:**

71 UR of patients with BD and 66 healthy controls (HC) underwent fMRI scanning while performing an emotion regulation task. Hierarchical cluster analysis was performed on extracted signal change during emotion down-regulation in pre-defined regions of interest (ROIs). Identified subgroups were compared on neural activation, demographic, clinical, and cognitive variables.

**Results:**

Two subgroups of UR were identified: subgroup 1 (39 UR; 55%) was characterized by *hypo*-activity in the dorsolateral, dorsomedial, and ventrolateral prefrontal cortex and the bilateral amygdalae, but comparable activation to HC in the other ROIs; subgroup 2 (32 UR; 45%) was characterized by *hyperactivity* in all ROIs. Subgroup 1 had lower success in emotion regulation compared to HC and reported more childhood trauma compared to subgroup 2 and HC. Subgroup 2 reported more anxiety, lower functioning, and greater attentional vigilance toward fearful faces compared to HC. Relatives from both subgroups were poorer in recognizing positive faces compared to HC.

**Conclusions:**

These findings may explain the discrepancy in earlier fMRI studies on emotion regulation in UR, showing two different subgroups of UR that both exhibited aberrant neural activity during emotion regulation, but in opposite directions. Furthermore, the results suggest that impaired recognition of positive facial expressions is a broad endophenotype of BD.

## Introduction

Bipolar disorder (BD) is a severe and chronic psychiatric disorder that affects mood, energy, and ability to function due to recurring episodes of depression and (hypo)mania (American Psychiatric Association, 2013). The estimated global prevalence of BD is 1–2% (Moreira, Van Meter, Genzlinger, & Youngstrom, 2017), ranking among the most disabling diseases worldwide (Vigo, Thornicroft, & Atun, 2018). The exact pathogenesis of BD is still largely unknown, but twin studies have revealed high heritability estimates for BD of about 60–85% (Smoller & Finn, 2003). Consequently, unaffected first-degree relatives (UR) of BD patients have a 10-fold increased risk of developing the disorder compared to individuals without familial liability for BD (Smoller & Finn, 2003). UR also has an elevated susceptibility to other psychiatric disorders, partially due to the shared genetic vulnerability with other psychiatric illnesses such as schizophrenia and major depression (Gordovez & McMahon, 2020). Despite this, we have little insight into *who* is at risk of eventually developing psychiatric illness.

A way to increase the ability to predict psychopathology in UR is by identifying endophenotypes in BD (Hasler et al., 2006). Endophenotypes are heritable, state-independent traits, that are related to the illness (Gottesman & Gould, 2003). They reflect biological traits that are expressions of underlying genetic factors (Guglielmo, Miskowiak, & Hasler, 2021) and are thus present in both ill and remitted patients, as well as in their UR at a higher rate than in the general population (Leboyer et al., 1998). Accordingly, the identification of BD endophenotypes can potentially help predict illness onset in at-risk individuals and thus provide a platform for the development of prophylactic interventions. This is highly needed since the success of finding such predictive biomarkers has been not successful thus far. Moreover, endophenotypes could enhance our understanding of the underlying pathophysiology and heterogeneous symptomatology in BD patients, leading to greater diagnostic accuracy and eventually a better prognosis of the disease.

The severe fluctuations in mood and affective lability that are characteristic of BD are associated with impaired emotion regulation (Oliva et al., 2023; Townsend & Altshuler, 2012). In healthy individuals, effective emotion regulation relies on the interactive coupling between prefrontal cortical (PFC) brain regions that are involved in cognitive control (i.e., the dorsolateral prefrontal cortex (DLPFC), the dorsomedial prefrontal cortex (DMPFC), and the ventrolateral prefrontal cortex (VLPFC)) and limbic brain regions involved in emotional processing, especially the amygdala (Berboth & Morawetz, 2021). During emotion, downregulation of negative emotions using cognitive reappraisal, increased activation of these PFC regions exerts a top-down inhibitory influence on the amygdala (Banks et al., 2007; Buhle et al., 2014). In BD patients, however, aberrant neural activation and connectivity have been found in this fronto-limbic network during voluntary emotion regulation in depressive, manic, and remitted states (Kurtz et al., 2021; Townsend & Altshuler, 2012). Although UR appears to have no impairment in regulating their emotions at the behavioral level (Kjærstad et al., 2020), functional magnetic resonance imaging (fMRI) studies of neural activations in UR revealed aberrations in similar brain regions as observed in BD patients (i.e., in the VLPFC, DMPFC, and DLPFC; Miskowiak et al., 2017). This discrepancy between behavioral and neural findings is partly due to abnormal neuronal responses being a more sensitive assay of aberrant brain function than overt behavioral responses. Indeed, self-report measures employed during fMRI may not be sufficient to detect subtle group differences between UR and HC, and the reliability of self-report ratings may be influenced by various biases, such as social desirability effects (Zilverstand, Parvaz, & Goldstein, 2017). Yet, results comparing UR and healthy controls (HC) are conflicting, with some studies indicating decreased amygdala activity (Kanske, Schönfelder, Forneck, & Wessa, 2015), reduced PFC activity (Meluken et al., 2019), or no neural abnormalities (Kjaerstad et al., 2021).

This discrepancy in findings across fMRI studies of UR could reflect the heterogeneity among UR in emotion regulation abilities. This would be consistent with the emerging evidence for emotional cognition subgroups among patients with BD, as identified both behaviorally (de Siqueira Rotenberg et al., 2023; Varo et al., 2021) and at a neural level (Kjærstad et al., 2022; Njau et al., 2020). At a neural level, two fMRI studies investigated subgroups of BD patients during the down-regulation of negative emotions (Kjærstad et al., 2022; Njau et al., 2020). The subgroups found by Njau et al. (2020) were characterized by either (1) hypoactivation in the entire emotion regulation network, but slightly increased activation in the VLPFC and subgenual cingulate (33%), or by (2) lower activation in the amygdala and increased wider-spread activation in PFC regions, especially the DLPFC (67%). More hospitalizations for depression and later onset of manic episodes were observed in this first subgroup compared to the second subgroup. Our group also identified two neuronal subgroups (Kjærstad et al., 2022). However, these were marked by either (1) heightened activity of the amygdala and normal PFC activity (75%), or (2) broad hypoactivation in both the limbic and PFC regions (25%), both compared to the other subgroup. Patients in this second subgroup had a history of more and longer mixed episodes.

Yet, no study to date has investigated emotion regulation subgroups based on neural activity in UR of BD patients. The presence of trait-related abnormalities in neural responses during emotion regulation in both BD patients and their UR could present a promising endophenotype for BD (Miskowiak et al., 2017). The current study aimed to examine whether discrete subgroups of UR could be identified based on their neural activity during emotion regulation and whether these groups differ on clinical, demographic, and cognitive variables. Based on previous studies in the patient population, we hypothesized that two different subgroups would be identified: one characterized by aberrant neural activity during emotion regulation, specifically PFC hypo-activity, and another characterized by comparable activation to HC.

## Methods

### Study design

This cross-sectional study is embedded in the ongoing and longitudinal Bipolar Illness Onset (BIO)-study (Kessing et al., 2017). For the present report, baseline data of UR of recently diagnosed BD patients and HC was investigated. Data collection of the current sample took place from February 2017 to February 2021. *The authors assert that all procedures contributing to this work comply with the ethical standards of the relevant national and institutional committees on human experimentation and with the Helsinki Declaration of 1975, as revised in 2008.* Ethical approval for the BIO study was given by the Committee on Health Research Ethics of the Capital Region of Denmark (protocol number: H-7-2014-007) and the Danish Data Protection Agency, Capital Region of Copenhagen (protocol number: RHP-2015-023). Informed consent of all participants was obtained prior to study participation.

### Participants

Patients with BD were recruited from the Copenhagen Affective Disorder Clinic, Psychiatric Centre Copenhagen, Denmark, where they had received a first diagnosis of BD within two years prior to study enrolment. Upon consent of patients, their first-degree relatives (i.e., patients’ siblings or offspring) 17 years of age or older were invited to participate in the study as well. Relatives were excluded in case of a personal lifetime history or current diagnosis of treatment-required psychiatric illness, which was confirmed by MDs or MSc in psychology using a semi-structured interview based on the Schedules for Clinical Assessment in Neuropsychiatry (SCAN; Wing et al., 1990). Age- and sex-matched HC without personal or familial history of mental disorders were recruited from the University Hospital Blood Bank, Rigshospitalet, Copenhagen. Mood symptoms were rated using the Hamilton Depression Rating Scale-17 (HDRS; Hamilton, 1967) and the Young Mania Rating Scale (YMRS; Young, Biggs, Ziegler, & Meyer, 1978). General exclusion criteria for both UR and HC were total scores >14 on HDRS or YMRS, contraindications for MRI (e.g., metal implants, pregnancy, etc.), a history of brain injury, neurological disorders including dementia, current severe somatic illness, and current substance abuse disorder. All participants were fluent in Danish.

## Measures

### Emotion regulation paradigm

All participants underwent fMRI scanning while performing a well-established emotion regulation paradigm (Banks et al., 2007; Phan et al., 2005), which has previously been shown to activate emotion regulation networks (Kjaerstad et al., 2021). During this task, participants were presented with 24 neutral and 48 unpleasant pictures from the International Affective Picture System (IAPS) (Lang, Bradley, & Cuthbert, 1997), which were shown in sets of four images sorted per category. For every set, they were asked to either passively view (“view”) the images or to voluntarily downregulate their emotional response to the unpleasant images (“dampen”). Subsequently, they had to rate the level of unpleasantness on a 5-point Likert scale (from 1 ‘not at all unpleasant’ to 5 ‘very unpleasant’) by pressing a button on a response box with their right hand. The three conditions (i.e., passive view neutral, passive view unpleasant, and downregulate unpleasant) were each shown randomly six times. After the instruction “view” or “dampen” (4 s), a set of images was shown (4 s per image), and participants had 4 s to respond to the Likert scale. A fixation cross on a black blank screen was shown for 16 s before the next set of images appeared. The total time of the task was 12 minutes (Supplementary Figure S1). The sets of unpleasant images in the “view” and “dampen” conditions comprised different images, but those were matched for valence (*p* = .54) and arousal (*p* = .56) consistent with the normative ratings of IAPS (Lang, Bradley, & Cuthbert, 1997). No instructions were given to the participants about what emotion regulation strategy to use during the task, to allow them to use the same strategies they habitually employ and increase the ecological validity of the paradigm. However, after participants had completed the fMRI task, they were asked in an open question whether there was a particular strategy they had used. According to their descriptions, two independent researchers categorized how often each participant had mentioned a particular strategy (Supplement C).

### Measures of mood, functioning, quality of life, and childhood trauma

Mood ratings were conducted with the HDRS and YMRS to assess subsyndromal depressive and mania symptoms, respectively. The two anxiety items in the HDRS (i.e., items 10 and 11) were used to assess somatic and psychological anxiety symptoms. Overall functioning during the recent 14 days was assessed by the 24-item semi-structured interview Functioning Assessment Short Test (FAST; Rosa et al., 2007). The FAST assesses disability or impairment across six distinct domains of functioning, which include autonomy, occupational performance, cognitive abilities, financial matters, interpersonal relationships, and leisure activities, and has a cut-off score of >11. Quality of life was rated by the European Quality of Life 5 Domain (EQ-5D; The EuroQol Group, 1990), comprising the dimensions of mobility, self-care, usual activities, pain/discomfort, and anxiety/depression. Raw scores were converted to index scores based on Danish norms. To assess childhood trauma the Childhood Trauma Questionnaire (CTQ; Bernstein, Fink, Handelsman, & Foote, 1998) was used, which included the subdomains physical abuse, emotional abuse, sexual abuse, emotional neglect, and physical neglect.

### Measures of emotional and non-emotional cognition

Emotional cognition was tested using various paradigms. The Social Scenarios Task assessed emotion reactivity and regulation of social scenarios (Kjærstad et al., 2016). The Facial Expression Recognition Test (FERT) was used to evaluate participants’ ability to identify facial expressions of the six basic emotions (i.e., anger, disgust, fear, happiness, sadness, and surprise) that morphed at 10% intensity levels ranging from neutral faces to the full emotion (Harmer, Shelley, Cowen, & Goodwin, 2004). Faces were from Ekman and Friesen’s Pictures of Facial Affect series (Ekman, 1976). Four images per intensity level for every emotion, including a neutral face, were presented in random order, totaling 250 faces shown for 500 ms each followed by a blank screen. The accuracy of the identification and reaction times were measured. The Dot Probe Test was used to assess attentional vigilance towards emotional faces (Murphy, Downham, Cowen, & Harmer, 2008).

Non-emotional cognition was assessed using a large neuropsychological test battery that tested cognitive functions in the domains of attention, verbal learning, working memory, and executive functioning, resulting in a total score that was formulated as a global cognition score (for details see Kjærstad et al., 2019).

Given our aim is to assess how URs deviate from the HC group regulation, we Z-transformed raw scores for both the emotional and non-emotional tests using the HC group’s means and standard deviations (SD). Truncation of the z-scores was performed at the threshold of −4 or + 4 to prevent extreme scores from unduly influencing the analysis.

## Analyses

### fMRI data analysis

See Supplement A for fMRI data acquisition. For both pre-processing of the data and conducting the first-level analysis the fMRI Expert Analysis Tool (FEAT) version 6.0 (Woolrich, Ripley, Brady, & Smith, 2001) from the FMRIB Software (FSL; http://www.fmrib.ox.ac.uk/fsl) was used. Pre-processing of the data consisted of brain extraction, correction of the B0 field distortion based on the field map image, motion correction, linear and nonlinear registration to structural space, spatial normalization to the Montreal Neurological Institute (MNI) standard space, and spatial smoothing (Gaussian kernel full-width half maximum = 5 mm). The registrations of all participants were visually inspected to ascertain a good fit. In each session, the time series were high pass-filtered to min 0.008 Hz. For the first-level analysis, a general linear model (GLM) was conducted for the three different conditions: ‘passive view neutral’, ‘passive view unpleasant’, and ‘downregulate unpleasantly’. These conditions were modeled as blocks convolved with a canonical hemodynamic response function, to which a temporal derivative was added. To calculate emotion regulation, which was our main contrast of interest, the difference between ‘downregulate unpleasant’ > ‘passive view unpleasant’, was taken. Furthermore, emotion reactivity was calculated for comparison purposes by contrasting ‘passive view negative’ > ‘passive view neutral’ (for results pertaining to emotion reactivity, see Supplementary Table S3).

To account for head movement the GLM model also included six standard motion parameters. No participant was excluded due to head movement, defined as a mean framewise displacement > .2 mm.

### Regions of interest

Eleven regions of interest (ROIs) were a priori selected based on findings of a previous meta-analysis on emotion regulation using IAPS images in healthy individuals (Supplementary Table S1) (Morawetz, Bode, Derntl, & Heekeren, 2017). The ROIs were each composed of 10 mm spheres and were constructed in FSLeyes, comprising the bilateral inferior frontal gyri/VLPFC (Brodmann area [BA] 47), bilateral middle frontal gyri/DLPFC (BA 6/8/10), bilateral superior frontal gyri/DMPFC/DLPFC (BA 6/9), bilateral angular gyri (BA 40/39), left middle temporal gyrus (BA 21) and posterior cingulate cortex (BA 23). Additionally, the anatomically defined left and right amygdalae based on the probabilistic Harvard-Oxford subcortical structural atlas with a threshold of 30% were extracted (Desikan et al., 2006). For each participant, the mean percent BOLD signal change of all eleven ROIs during both the contrasts of emotion regulation (‘downregulate unpleasant’ > ‘passive view unpleasant’) and emotion reactivity (‘passive view negative’ > ‘passive view neutral’) were extracted using the featquery tool in FSL.

### Statistical analyses

All statistical analyses were performed in IBM Statistical Package for Social Sciences (SPSS) version 28. Behavioral in-scanner ratings were arcsine transformed and emotion regulation success was calculated by subtracting unpleasantness ratings during ‘downregulate unpleasant’ conditions from ‘passive view unpleasant’ conditions, whereas emotion reactivity was calculated by subtracting unpleasantness ratings from ‘passive view neutral’ conditions from ‘passive view negative’ conditions. In accordance with previous studies using hierarchical cluster analysis to investigate homogeneous subgroups of patients with BD (e.g., Njau et al., 2020), we conducted an agglomerative hierarchical cluster analysis (HCA) on the extracted mean percent BOLD signal changes of the emotion regulation contrast to identify homogeneous subgroups of UR based on their neural responses during emotion regulation. The similarity between cases was determined with squared Euclidian Distance and Ward’s linkage as an agglomeration procedure (see Supplement B for more information on the selection of cluster solutions. The generated dendrogram was visually inspected to retain the optimal number of clusters. To test the validity of the clustering solutions, discriminant function analyses using leave-one-out classifications were performed.

To get insight into the characteristics of the identified subgroups, we compared the resulting subgroups and HC on their neural activation per ROI, and their emotional and non-emotional cognitive performance, as well as clinical and demographic variables (e.g., age, sex, years of education, success of behavioral in-scanner emotion regulation, and scores on the HDRS total, HDRS anxiety items, YMRS total, FAST total, FAST interpersonal relationships, EQ5D index, and CTQ total). The mean percent BOLD signal change of the identified neuronal subgroups and HC were compared per ROI using analysis of variance (ANOVA) with Sidak correction for multiple comparisons. Furthermore, clinical, demographic, and cognitive variables were tested for data normality distribution using the Shapiro–Wilk test. We employed ANOVA with Sidak correction for multiple comparisons for normally distributed variables (years of education, IQ, non-emotional cognitive variables, reaction time on the FERT). The non-parametric Kruskal-Wallis test was used for all other variables, which were not normally distributed. Pearson’s chi-square (χ^2^) was used for categorical variables (sex, smoking). Post-hoc analyses were conducted to investigate pairwise comparisons. The threshold level for statistical significance was set at *p* < .05 (two-tailed).

The BOLD signal change during downregulation was averaged for the ROIs within the (1) bilateral amygdalae; (2) PFC (mean signal change from the following extracted ROIs: bilateral VLPFC, DLPFC, DMPFC); and (3) temporoparietal regions (mean average of bilateral angular gyri and left middle temporal gyrus). The ROIs were grouped to reflect the distinct neural areas involved in emotion regulation (e.g., prefrontal cortex activation associated with cognitive control and brain activation of subcortical regions associated with emotion generation) as well as to reduce the number of comparisons. We conducted exploratory Pearson’s correlations within each subgroup to examine associations between the averaged BOLD signal in these regions and the variables that were statistically significantly different from the other subgroups.

Given that the risk of illness onset may decrease with older age, we conducted a post-hoc subgroup analysis investigating differences in neural activity in the pre-defined emotion regulation network between young UR (< 25 years of age) and adult UR (≥ 25 years of age) in the resulting subgroups, respectively, using independent samples t-tests. This age cut-off was selected to distinguish between young UR (i.e., potential markers of risk) and adult UR (i.e., potential markers of resilience), in accordance with our previous report (Coello et al., 2024).

## Results

### Participants

Our original sample consisted of 72 UR and 66 HC. Out of these, one UR was excluded due to a previous depression and a current diagnosis of generalized anxiety disorder and receiving psychotropic medication. Therefore, the current sample consisted of a total of 137 participants, of which 71 UR and 66 HC (Supplementary Table S2).

### Neuronal subgroups during emotion regulation

The agglomerative HCA with Ward’s linkage measure and squared Euclidian distance revealed an optimal clustering of two subgroups among the UR based on their neural activity in the emotion regulation network during the voluntary downregulation of negative emotions (see Supplementary Figures S2 and S3 for the resulting dendrogram and visual representation of the agglomeration matrix). Subgroup 1 consisted of 39 UR (55%) and subgroup 2 of 32 UR (45%). To assess the validity of the clustering outcomes of the HCA, a discriminant function analysis was performed, which showed a discriminant function with a Wilks’ λ = .353, χ^2^ (13) = 65.971, *p* < .001. According to the DFA, 95.8% of all UR were correctly classified into clusters by the HCA. The mean percent BOLD signal change (in the emotion downregulation contrast) in the left superior frontal gyrus in the DMPFC contributed the most to clustering (r = .67).

## Comparisons between identified subgroups of unaffected relatives and healthy controls

### Neural activity within the emotion regulation network

**Figure 1.**
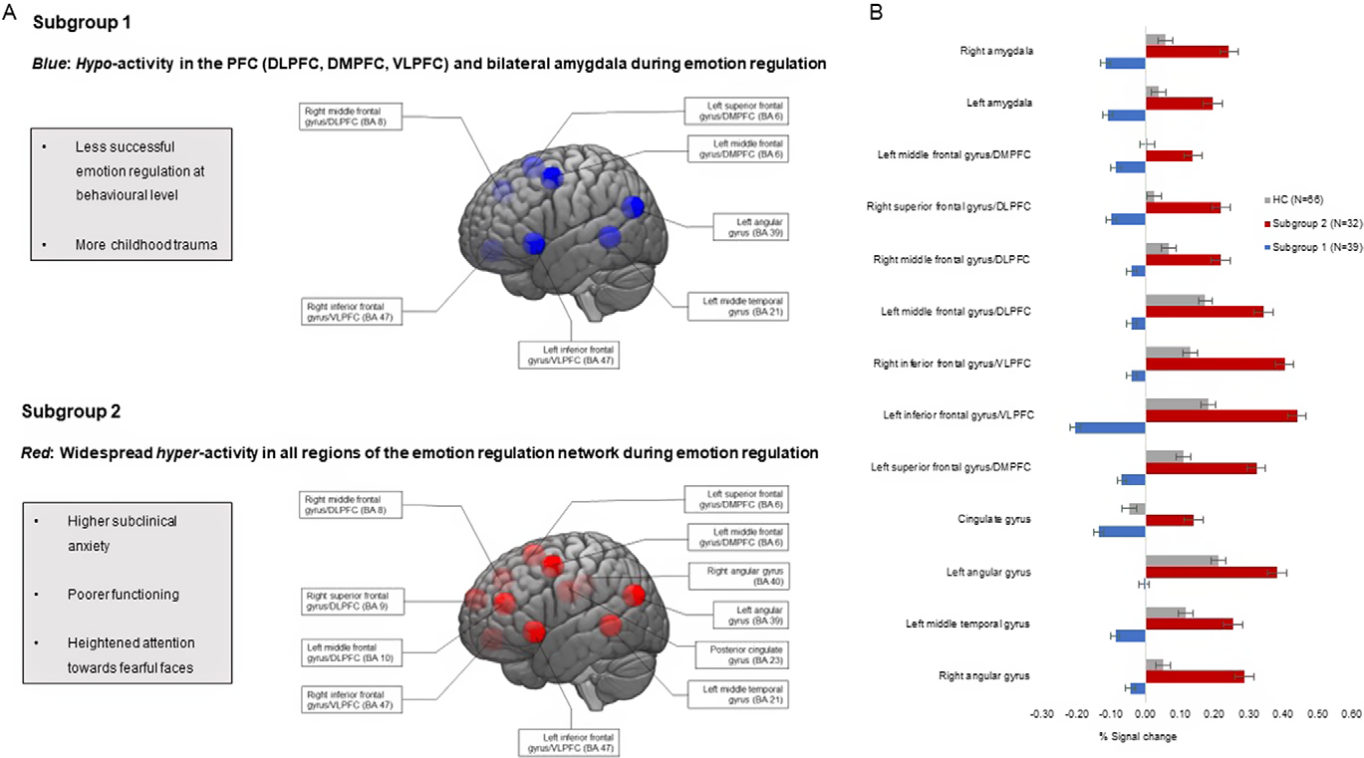
(a) Eleven regions of interest (ROI) in the emotion regulation network in the brain, of which the mean percent Blood Oxygen Level Dependent signal change was recorded during emotion downregulation. Top: During emotion regulation, unaffected relatives in subgroup 1 presented with hypo-activity in the left superior frontal gyrus in the dorsomedial prefrontal cortex (DMPFC), bilateral inferior frontal gyrus in the ventrolateral prefrontal cortex (VLPFC), bilateral middle frontal gyrus in the dorsolateral prefrontal cortex (DLPFC), left middle temporal gyrus and angular gyrus, as well as bilateral amygdalae, but were comparable to controls in the right angular gyrus, cingulate gyrus, right superior frontal gyrus in the DLPFC, and left middle frontal gyrus in the DMPFC. Bottom: Subgroup 2 showed significant hyperactivity in all ROIs of the emotion regulation network compared to controls. (b) Visual representation of the extracted signal change of the neural activation for all regions of interest during voluntary emotion downregulation of negative emotions for the two subgroups of unaffected relatives of patients with bipolar disorder and healthy controls. Error bars represent the standard error of the mean.

Analyses investigating the mean percent BOLD signal change of all ROIs within the emotion regulation network during the emotion regulation paradigm showed a statistically significant difference for all regions across the different clusters of UR and HC (all *p*-values < .001). Pairwise comparisons between subgroup 1 and subgroup 2 showed statistically significant differences for all ROIs (all *p*-values < .001). When comparing the subgroups to HC, subgroup 1 showed *hypo*-activity in the left superior frontal gyrus in the DMPFC, bilateral inferior frontal gyrus in the VLPFC, bilateral middle frontal gyrus in the DLPFC, left middle temporal gyrus and angular gyrus, as well as bilateral amygdalae (*p*-values ≤ .04), but were comparable to HC in the right angular gyrus, cingulate gyrus, right superior frontal gyrus in the DLPFC, and left middle frontal gyrus in the DMPFC (*p*-values ≥ .08). On the other hand, subgroup 2 showed significant *hyper-*activity in all ROIs compared to HC (*p*-values ≤ .02) (Table 1, Figure 1 and Supplementary Figure S4).

**Table 1.** Three-way and pairwise comparisons of neural activation in the emotion regulation network during emotion regulation (‘downregulate unpleasant’ > ‘passive view unpleasant’ contrast) for the two subgroups of unaffected relatives of patients with bipolar disorder and healthy controls (HC). Bold text indicates significant values of p < .05.

	Means				Three-way comparisons, p-value	Pairwise comparisons, p-value		
	Subgroup 1 (N = 39)	Subgroup 2 (N = 32)	HC (N = 66)	F		SG1 vs HC	SG 2 vs HC	SG 1 vs SG 2
Right angular gyrus	−0.04	0.29	0.05	12.15	**<.001**	.290	**<.001**	**<.001**
Left middle temporal gyrus	−0.09	0.26	0.12	19.66	**<.001**	**<.001**	**.021**	**<.001**
Left angular gyrus	0.00	0.38	0.21	20.08	**<.001**	**<.001**	**.008**	**<.001**
Cingulate gyrus	−0.14	0.14	−0.05	16.90	**<.001**	.082	**<.001**	**<.001**
Left superior frontal gyrus/DMPFC	−0.07	0.32	0.11	26.36	**<.001**	**<.001**	**<.001**	**<.001**
Left inferior frontal gyrus/VLPFC	−0.20	0.44	0.18	22.95	**<.001**	**<.001**	**.011**	**<.001**
Right inferior frontal gyrus/VLPFC	−0.04	0.40	0.13	17.09	**<.001**	**.027**	**<.001**	**<.001**
Left middle frontal gyrus/DLPFC	−0.04	0.34	0.17	19.45	**<.001**	**<.001**	**.009**	**<.001**
Right middle frontal gyrus/DLPFC	−0.04	0.22	0.07	12.88	**<.001**	**.038**	**.005**	**<.001**
Right superior frontal gyrus/DLPFC	−0.10	0.22	0.02	11.82	**<.001**	.077	**.004**	**<.001**
Left middle frontal gyrus/DMPFC	−0.09	0.14	0.01	9.28	**<.001**	.116	**.017**	**<.001**
Left amygdala	−0.11	0.20	0.04	19.36	**<.001**	**.001**	**.002**	**<.001**
Right amygdala	−0.12	0.24	0.06	26.15	**<.001**	**<.001**	**<.001**	**<.001**

Abbreviations: DMPFC = dorsomedial prefrontal cortex; VLPFC = ventrolateral prefrontal cortex; DLPFC = dorsolateral prefrontal cortex; SG = subgroup.

### Behavioral ratings of emotion downregulation

**Figure 2.**
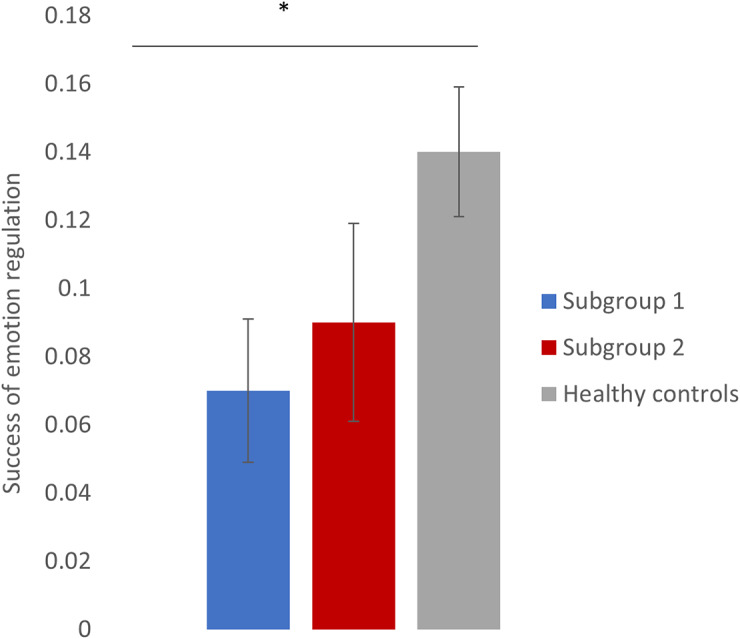
Mean behavioral success of emotion regulation based on in-scanner ratings during the emotion regulation paradigm for the two subgroups of UR and HC. Subgroup 1 was significantly poorer at downregulating their emotional responses to aversive images compared to HC. No statistically significant difference was found between subgroup 2 and HC, nor with subgroup 1. Error bars represent the standard error of the mean. The significance level was set at p < .05 with Sidak correction.

The success of emotion regulation based on the in-scanner behavioral ratings was different across the groups (χ^2^(2) = 6.40, *p* = .04). This statistically significant difference was driven by subgroup 1 performing significantly worse than HC (*p* = .02). Although subgroup 2 scored lower than HC on their success in emotion regulation, no statistically significant difference was found between subgroup 2 and HC (*p* = .07), nor between subgroup 1 and 2 (*p* = .85) (Figure 2 and Supplementary Figure S5).

### Clinical and demographic variables

The two subgroups of UR were comparable to HC in age, sex, years of education, IQ, subsyndromal mood symptoms, BMI, smoking status, and quality of life. There was a statistically significant difference between the three groups in childhood trauma (*p* < .001) with subgroup 1 reporting higher rates of childhood trauma than both subgroup 2 (*p* = .04) and HC (*p* < .001), specifically within the domains of emotional and physical neglect. In contrast, the significant group difference in anxiety items of the HDRS (*p* = .006) was driven by subgroup 2 experiencing more anxiety compared to HC (*p* = .003), whereas no significant difference was found when comparing to subgroup 1 (*p* = .10). Furthermore, results revealed a significant difference between the three groups in total functioning (i.e., total FAST) (*p* < .001), as well as in interpersonal relations (*p* = .002), both of which were driven by subgroup 2 having significantly more functional impairments compared to both HC (*p*-values ≤ .003) and subgroup 1 (*p*-values ≤ .02). Finally, a significant group difference was found for alcohol (*p* = .004), driven by HC consuming more units of alcohol compared to both UR subgroups (*p*-values ≤ .004), with no statistical difference between the two UR subgroups (*p* = .35) (Table 2).

**Table 2.** Comparisons of clinical and demographic variables across the two subgroups of unaffected relatives of patients with bipolar disorder and healthy controls.

	Means (SD)				Three-way comparisons, p-value	Pairwise comparisons, p-value		
	Subgroup 1 (N = 39)	Subgroup 2 (N = 32)	HC (N = 66)	F/H/χ^2^		SG 1 vs HC	SG 2 vs HC	SG 1 vs SG 2
Age	28.28 (6.92)	26.72 (7.16)	29.59 (9.55)	1.96	0.37			
Sex, (n % female)	20 (53%)	17 (53%)	41 (62%)	1.42	0.49			
Years of education	15.04 (2.82)	15.28 (2.75)	15.71 (2.26)	0.92	0.40			
IQ	111.25 (6.86)	109.85 (6.41)	112.74 (4.76)	2.71	0.07			
HDRS, total score	1.10 (1.68)	1.81 (2.51)	1.00 (1.16)	1.50	0.47			
HDRS, anxiety items	0.03 (0.16)	0.16 (0.45)	0.00 (0.00)	10.07	**0.006**	0.19	**0.003**	0.10
YMRS, total score	1.13 (1.79)	0.41 (1.04)	0.76 (1.71)	5.66	0.06			
FAST, total score	1.87 (2.47)	5.88 (8.72)	1.15 (1.67)	14.59	**<.001**	0.14	**<.001**	**0.02**
FAST, interpersonal relationships	0.24 (0.63)	1.12 (1.96)	0.26 (.56)	12.07	**0.002**	0.64	**0.003**	**0.004**
Quality of life, EQ–5D index	0.96 (0.07)	0.97 (.07)	0.97 (.06)	0.81	0.67			
CTQ Total Score	32.46 (5.41)	30.75 (7.79)	28.31 (4.08)	17.44	**<.001**	**<.001**	0.23	**0.04**
CTQ Physical Abuse	5.08 (0.36)	5.38 (1.62)	5.08 (0.51)	1.74	0.42			
CTQ Emotional Abuse	6.30 (2.00)	6.56 (2.20)	5.66 (1.29)	5.93	0.05			
CTQ Sexual Abuse	5.11 (0.66)	5.28 (1.42)	5.09 (0.52)	0.51	0.78			
CTQ Emotional Neglect	8.68 (3.12)	7.16 (2.76)	6.59 (2.37)	13.35	**0.001**	**<.001**	0.52	**0.02**
CTQ Physical Neglect	7.30 (2.72)	6.38 (2.73)	5.98 (1.72)	7.88	**0.02**	**0.008**	0.99	**0.04**
BMI	24.56 (4.60)	23.38 (2.56)	23.50 (2.84)	1.38	0.50			
Currently smoking, n (%) yes	8 (21%)	2 (7%)	13 (20%)	2.95	0.23			
Alcohol, units per week	3.67 (4.50)	5.92 (6.65)	6.02 (4.19)	10.87	**0.004**	**<.001**	**0.004**	0.35
Success of emotion regulation during the in-scanner task (IAPS)	0.07 (0.13)	0.09 (0.16)	0.14 (0.16)	6.40	**0.04**	**0.02**	0.07	0.85

*Note:* Continuous variables were non-normally distributed and analyzed with non-parametric tests (e.g., Kruskal-Wallis H), with the exception of ‘years of education’ and ‘IQ’, which were analyzed using parametric tests. Bold text in the table indicates significant values (*p* < .05).

Abbreviations: SD = standard deviation; SG 1 = subgroup 1; SG 2 = subgroup 2; HC = healthy controls; HDRS=Hamilton Depression Rating Scale; YMRS=Young Mania Rating Scale; IAPS=International Affective Picture System; FAST = Functioning Assessment Short Test; EQ-5D = European Quality of life – 5 Dimensions.; CTQ = Childhood Trauma Questionnaire; BMI=Body Mass Index.

### Emotional and non-emotional cognition

Regarding emotional cognition, results revealed statistically significant differences between the three groups in recognition accuracy of positive faces (*p* < .001). This difference was driven by subgroups 1 and 2 being less accurate when recognizing positive faces (happy, surprise) compared to HC (*p*-values ≤ .02), with no significant difference between the two UR subgroups (*p* = .23). In addition, there was a statistically significant difference between the three groups in attentional inference of explicitly presented fearful faces (*p* = .03), driven by subgroup 2 exhibiting more attentional vigilance towards fearful faces compared to HC (*p* = .01), with no significant differences between subgroup 1 and HC or the two subgroups (*p*-values ≥ .08).

Regarding non-emotional cognition (i.e., global cognition, processing speed, attention, verbal learning, working memory, and executive function), analyses revealed no statistically significant differences between the two subgroups of UR and HC. There was a trend towards a statistically significant group difference in working memory and executive functioning, driven by poorer performance in both subgroups of UR compared to HC (*p* = .07) (Table 3).

**Table 3.** Comparisons of emotional and non-emotional cognition across the two subgroups of unaffected relatives of patients with bipolar disorder and healthy controls.

	Means (SD)				Three-way comparisons, p-value	Pairwise comparisons, p-value		
	Subgroup 1 (N = 39)	Subgroup 2 (N = 32)	HC (N = 66)	F/H		SG 1 vs HC	SG 2 vs HC	SG 1 vs SG 2
**Non-emotional cognition**								
Global cognition	−0.12 (0.56)	−0.13 (0.36)	0.00 (0.56)	1.02	0.36			
Processing speed	−0.17 (0.59)	−0.27 (0.62)	0.00 (0.72)	1.97	0.14			
Attention	0.18 (0.98)	0.01 (0.61)	0.00 (0.87)	0.64	0.53			
Verbal learning	−0.32 (1.16)	0.00 (0.65)	0.00 (0.86)	1.71	0.19			
Working memory and executive function	−0.19 (0.52)	−0.25 (0.51)	0.00 (0.61)	2.68	0.07			
**Emotional cognition**								
**Social Scenarios Test**								
Emotion reactivity - neutral	0.28 (1.12)	0.40 (1.13)	−0.02 (0.92)	5.20	0.07			
Emotion reactivity - negative	0.00 (1.06)	−0.18 (1.21)	0.00 (1.00)	0.66	0.72			
Emotion reactivity - positive	−0.59 (1.47)	−0.28 (1.47)	0.00 (1.00)	3.99	0.14			
Emotion downregulation – negative	−0.09 (0.83)	−0.45 (0.93)	0.00 (1.00)	2.47	0.09			
Emotion downregulation – positive	−0.19 (1.04)	−0.14 (1.02)	0.00 (1.00)	0.57	0.57			
**Facial Expression Recognition Test**								
Accuracy (d’), negative faces	−0.40 (1.29)	−0.31 (0.89)	0.00 (1.00)	1.97	0.14			
Accuracy (d’), positive faces	−0.49 (1.20)	−0.78 (0.87)	0.00 (1.00)	6.93	**<.001**	**0.02**	**<.001**	0.23
Speed, negative faces	0.18 (0.65)	0.18 (1.03)	0.00 (1.00)	1.36	0.51			
Speed, positive faces	0.29 (0.66)	0.27 (0.92)	0.00 (1.00)	3.88	0.14			
**Faces Dot-Probe Task**								
Masked fear	−0.13 (0.97)	−0.20 (1.29)	0.00 (0.98)	3.03	0.22			
Masked happy	−0.10 (0.54)	−0.29 (1.04)	−0.02 (0.88)	2.45	0.29			
Unmasked fear	0.30 (0.85)	0.50 (0.69)	0.00 (1.00)	7.33	**0.03**	0.08	**0.01**	0.56
Unmasked happy	−0.06 (0.34)	−0.17 (0.46)	−0.01 (0.98)	0.77	0.68			

*Note:* All scores are z-transformed. Cognitive variables were non-normally distributed and analyzed with non-parametric tests (e.g., Kruskal-Wallis H), with the exception of ‘global cognition’, ‘processing speed’, ‘working memory and executive function’, ‘social scenarios – emotion downregulation negative’, and ‘facial expression recognition test – accuracy (d’) negative faces’, which were analyzed using parametric tests. Bold text in the table indicates significant values (p < .05).

Abbreviations: SD = standard deviation; SG 1 = subgroup 1; SG 2 = subgroup 2; HC = healthy controls.

### Associations between BOLD levels and emotional downregulation, functioning, childhood trauma, anxiety, and emotional cognition

Pearson’s correlations were performed within each subgroup between the three domains of ROIs (i.e., bilateral amygdalae, PFC, and temporo-parietal regions) and the several variables that were found to be different across the subgroups (i.e., childhood trauma, a behavioral measure of emotional downregulation, anxiety, functioning, and emotional cognition). No significant correlations were found between any of the ROIs and the different variables (Supplementary Tables S4and S5).

### Post-hoc subgroup analysis comparing young versus adult-unaffected relatives

For UR belonging to subgroup 1, posthoc analyses revealed no significant difference between young (< 25 years of age, N = 13) and adult UR (≥ 25 years of age, N = 26) in neural activity from the pre-defined ROIs during emotion regulation (p-values ≥ .09). For UR belonging to subgroup 2, there was a statistically significant difference between young UR (< 25 years of age, N = 15) and adult UR (≥ 25 years of age, N = 17) in right middle frontal gyrus/DLPFC activity during emotion regulation (t = 2.33, p = .03), driven continued hyper-activation in young UR but normalization of hyper-activity in adult UR in subgroup 2. There was no significant difference in neural activity in the other regions of interest (p-values ≥ .17).

## Discussion

In the present study, two subgroups of unaffected relatives (UR) were identified based on their neural activation during voluntary emotion regulation of negative emotions. The first subgroup of UR was characterized by *hypo-*activity in the PFC (i.e., DLPFC, DMPFC, and VLPFC) and the amygdala, but was otherwise comparable to HC. Individuals in this subgroup reported significantly lower success in downregulating their emotional responses at a behavioral level and showed higher levels of childhood trauma. On the other hand, the second subgroup of UR was characterized by widespread *hyperactivity* in all regions of the emotion regulation network, higher levels of subclinical anxiety, lower functioning, and heightened attention towards fearful faces compared to HC. Both subgroups were less accurate at identifying positive faces compared to HC.

Our findings are in line with our initial hypothesis of identifying two distinct subgroups among UR. However, contrary to our hypothesis, and previous literature on BD patients, in which we expected one subgroup to exhibit similar activation patterns to HC, both subgroups showed aberrant neural activity in the emotion regulation network. No study before has investigated neural subgroups in UR, but prior fMRI research has observed comparable subgroups in the *BD patient* populations (Kjærstad et al., 2022; Njau et al., 2020). Similar to what we have found in UR, BD patients were characterized by one subgroup showing *hyper-*activation and the other showing *hypo-*activation in fronto-limbic regions during emotion regulation of negative emotions (Kjærstad et al., 2022; Njau et al., 2020). Studies of the neural correlates of emotion regulation in UR *at a group level* have hitherto yielded incongruent outcomes (Kanske, Schönfelder, Forneck, & Wessa, 2015; Kjaerstad et al., 2021; Meluken et al., 2019). In accordance with the pattern of neural activity in subgroup 1, previous studies comparing UR of patients with BD and HC revealed hypo-activation in the amygdala (Kanske, Schönfelder, Forneck, & Wessa, 2015) and PFC (Meluken et al., 2019), whereas other studies found no such group-level neural abnormalities in UR (Kjaerstad et al., 2021). Our findings of two discrete subgroups of UR both of which show *abnormal* neural activity during emotion regulation compared to HC - albeit in distinct ways - may explain this discrepancy between studies, as well as the lack of significant behavioral differences between UR and HC in other studies (Kanske et al., 2013; Kjaerstad et al., 2021; Ladouceur et al., 2013). Notably, the hypo-activation of the DMPFC, DLPFC, and VLPFC in subgroup 1 closely resembles the consistent hypo-activity observed in these regions among BD patients (Kjærstad et al., 2022; Kurtz et al., 2021). These findings underline the heterogeneity of neural activity during emotion regulation not only within the BD patient population but also among their UR, emphasizing the diverse characteristics present in this group in line with the Research Domain Criteria (RDoC) framework (Cuthbert, 2014).

In line with our finding that both UR subgroups show abnormal (hypo- and hyper-) neural activity during emotion regulation, both subgroups also demonstrated a significantly lower tendency to use the emotion regulation strategy ‘cognitive reappraisal: distancing’ compared with HC. Successful employment of this emotion regulation strategy, i.e., adopting a new perspective or distancing oneself from the eliciting stimulus, has been associated with less negative affect as well as better interpersonal functioning and overall well-being (Picó-Pérez et al., 2017). In the context of our results, both neural *hyper*- and *hypo*-activity during attempts to down-regulate negative affect may underlie the reduced ability to adopt this emotion regulation strategy in URs.

The poorer emotion regulation coupled with *hypo*-activity of the PFC and amygdala as observed in subgroup 1 compared with HC may suggest inefficient recruitment of the emotion regulation network. Indeed, emotion regulation impairments in this subgroup seem to be driven by inefficient recruitment of the cognitive control network to down-regulate emotions, as evidenced by lower neural activity during active emotion regulation conditions compared to passive attend conditions (unlike UR in subgroup 2 and HC who show the opposite pattern, see Supplementary Figure S4). However, surprisingly, this group demonstrated better overall functioning than subgroup 2, despite having reported more childhood trauma, which is a well-established risk factor for BD (Quidé et al., 2020). This might reflect resilience in this subgroup of UR, suggesting that they have developed effective coping mechanisms (e.g., distraction during emotion regulation) to manage adverse emotional situations and thereby prevent illness onset, unlike their affected probands.

In contrast, subgroup 2 displays heightened activity in the emotion regulation network yet effectively downregulates their emotional response relative to HC. This suggests that subgroup 2 may require additional neuronal resources to manage their emotions effectively. Moreover, since earlier-onset BD appears to be associated with increased familial risk, it could be that older UR has surpassed the *peak risk* of illness onset. Hence, the normalization of DLPFC hyperactivity in this group may reflect signs of resilience in this subgroup of adult UR. On the other hand, heightened amygdala activity during emotion regulation has previously been associated with a greater risk of relapse in BD patients (de Siqueira Rotenberg et al., 2023), UR in subgroup 2 may be particularly susceptible to developing illness onset. As such, future longitudinal studies should investigate whether these differences in neural activity during emotion regulation in UR may act as predictors of onset or reflect resilience markers in UR subgroups. Indeed, whether hypo- and hyper-activity in the emotion regulation network are associated with resilience and risk of illness onset, respectively, will be investigated in the longitudinal segment of the ongoing BIO study (Kessing et al., 2017). Accordingly, this could facilitate earlier diagnosis, being associated with higher recovery rates, and form the basis for developing prophylactic interventions targeted to those at risk. Finally, examining the accuracy of identifying positive faces is warranted, as both subgroups performed significantly worse in this task compared to HC, possibly indicating a shared endophenotype in BD with a strong genetic basis.

The observation of aberrant neural activity in the subgroups of UR can inform clinical practice. Specifically, if the findings are replicated beyond our study, UR in subgroup 1 may particularly benefit from psychotherapy involving emotion regulation training and self-compassion, which has previously been shown to enhance the efficacy of cognitive reappraisal as an emotion regulation strategy (Diedrich, Hofmann, Cujipers, & Berking, 2016; Iwakabe, Nakamura, & Thoma, 2023). Subgroup 2 may benefit from a prophylactic CBT-based prevention program to reduce anxiety symptoms and improve overall functioning (Lawrence, Rooke, & Creswell, 2017).

The key strengths of the current study include the use of a well-defined sample of UR of newly diagnosed patients with BD and matched HC – both free of psychiatric illness. Furthermore, the study included a well-established fMRI paradigm to assess emotion regulation, as well as the use of a wide variety of both emotional and non-emotional cognitive assessments. Nevertheless, a few points should be kept in mind when interpreting the results. Firstly, due to limited financial resources, the sample undergoing fMRI was relatively small considering the subgrouping of UR, limiting the statistical power of the analyses performed. However, small sample sizes of N = 20 per subgroup have previously been found to yield sufficient statistical power using clustering algorithms (Dalmaijer, Nord, & Astle, 2022) and we have previously demonstrated significant subgroup differences using comparable samples of patients with BD (n = 87; (Kjærstad et al., 2022). Secondly, the study lacked follow-up data with regard to whether UR eventually developed a psychiatric illness. Thirdly, the cross-sectional design of the current study is insufficient to elucidate the longitudinal effects of the aberrant neural activity in the UR subgroups, but this remains to be investigated in the longitudinal part of the study. Lastly, while IAPS images are standardized, they may not fully capture the complexity of real-world emotional stimuli. Regulating emotions in response to these images may have limited ecological validity and may not accurately reflect how emotions are regulated in daily life.

In conclusion, our study revealed two distinct emotion regulation subgroups of UR of patients with BD, both showing aberrant neural activation compared to HC. These findings contribute to addressing the discrepancies between previous fMRI studies of emotion regulation in UR. It supports the hypothesis of impaired emotion regulation being a trait-related characteristic of BD. Moreover, impaired recognition of positive facial expressions is suggested to be a broader endophenotype, paving the way for a more thorough understanding of endophenotypes in BD. This will aid in building a foundation for developing prophylactic interventions for psychiatric illness in at-risk populations like relatives of patients with mood disorders.

## Supporting information

Kjærstad et al. supplementary material Kjærstad et al. supplementary material

## Supplementary material

The supplementary material for this article can be found at http://doi.org/10.1017/S0033291724003593.
