# Supplementary material for: Neural subgroups in unaffected first-degree relatives of patients with bipolar disorder during emotion regulation
Source: Psychol Med. 2025 Feb 12;55:e45. doi: 10.1017/S0033291724003593 (PMC12055023; doi:10.1017/S0033291724003593)
Supplement: Kjærstad et al. supplementary material [file S0033291724003593sup001.docx]

**Supplemental material**

**A. Methods**

*fMRI data acquisition*

Functional MRI scanning was performed at the Copenhagen University Hospital, Rigshospitalet using a 3-Tesla Siemens Prisma scanner and a 64-channel head-neck coil. A total of 366 blood oxygen dependent (BOLD) images were acquired during the emotion regulation paradigm with a T2*-weighted gradient echo spiral echo-planar (EPI) sequence and the following parameters: repetition time (TR) = 2 s; echo time (TE) = 30 ms; flip angle = 90°; 64 x 64 grid; field of view (FOV) = 230 x 230 mm; 32-slice volume; slice thickness = 3 mm, with 25% gaps in-between. The BOLD images were registered to T1-weighted structural images (TR = 1900 ms; TE = 2.58 ms; flip angle = 9°; distance factor = 50%; FOV = 230 × 230 mm; slice thickness = 0.9 mm). In addition, a standard B0 field map sequence was acquired with similar resolution as the fMRI sequence (TR = 400 ms; TE = 7.38 ms; flip angle = 60°) and used for geometric distortions correction of the BOLD images. Image quality was ensured by visual inspection of the individual images.


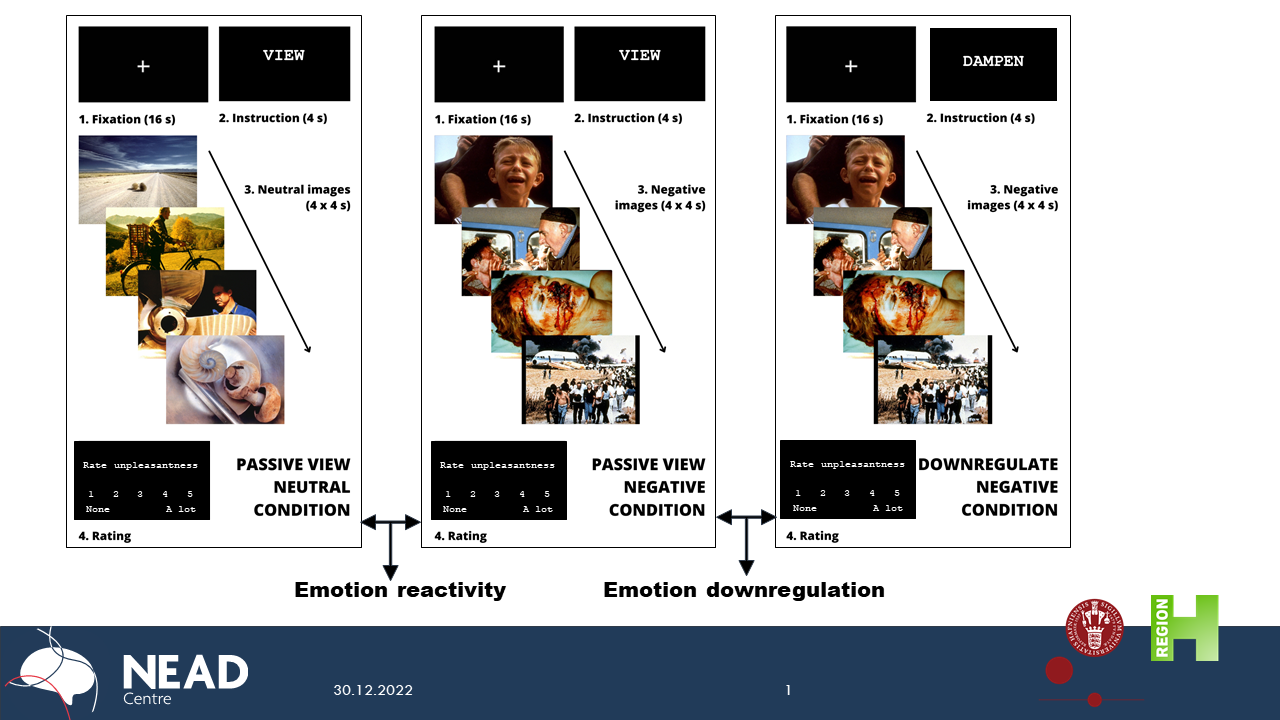


**Figure S1.** Emotion regulation paradigm performed during fMRI scanning, comprising (1) fixation, (2) instruction to either view or dampen, (3) viewing a series of four neutral or negative pictures, (4) behavioral rating of the experienced emotions on a 5-point Likert scale. The three different conditions were shown 6 times each in a random order.

**Table S1.** Regions of interest (ROIs) from Morawetz et al., (2017)

|  |  |  | MNI coordinates | | |
| --- | --- | --- | --- | --- | --- |
| Cluster # | Region | BA | x | y | z |
| 1 | Left superior frontal gyrus | 6 | -4 | 12 | 60 |
| 2 | Left inferior frontal gyrus | 47 | -46 | 26 | -8 |
| 3 | Right inferior frontal gyrus | 47 | 50 | 30 | -8 |
| 4 | Left angular gyrus | 39 | -54 | -60 | 24 |
| 5 | Left middle frontal gyrus | 6 | -42 | 8 | 48 |
| 6 | Right angular gyrus | 40 | 58 | -54 | 40 |
| 7 | Right middle frontal gyrus | 8 | 40 | 24 | 42 |
| 8 | Left middle temporal gyrus | 21 | -58 | -38 | -2 |
| 9 | Right superior frontal gyrus | 9 | 34 | 50 | 24 |
| 10 | Posterior cingulate gyrus | 23 | 0 | -20 | 30 |
| 11 | Left middle frontal gyrus | 10 | -34 | 48 | 16 |

**B. Results**

*Selection of optimal cluster solutions*

No study has previously investigated neural subgroups of unaffected relatives of patients with bipolar disorder during emotion regulation. Two studies have investigated neural subgroups of *patients* with bipolar disorder during emotion regulation (Kjærstad et al., 2022; Njau et al., 2020), both of which revealed two neural subgroups of patients. In the present study, inspection of the dendrogram (Figure S1) indicated support for two- and three-cluster solutions and the agglomeration schedule suggested that clustering should cease after the 68th stage, eliminating the last two stages (Figure S2; Yim & Ramdeen, 2015). Consequently, further exploration of two- and three-cluster solutions involved conducting discriminant functions analysis (DFA) for both clustering solutions. The DFAs indicated very high sensitivity for both two- and three-cluster solutions, with the same predictive power for both cluster solutions with 95.8% of originally grouped cases being correctly classified.

Further, inspection of the neural profiles during emotion regulation within the different cluster solutions showed that the two-cluster solution classified UR into a subgroup of UR (n=39) with hypo-activation in the left DMPFC, bilateral VLPFC, bilateral DLPFC, left middle temporal gyrus and angular gyrus, bilateral amygdalae, but otherwise comparable to controls, and a slightly smaller subgroup of UR (n=32) exhibiting widespread hyper-activity in the emotion regulation network during emotion regulation. The three-cluster solution was the same as the two-cluster solution, but with a small cluster containing only N=1 UR (i.e., three-clusters: Subgroup 1 N= 38; Subgroup 2 N=32, Subgroup 3 N=1).

In conclusion, analysis of the dendrogram, agglomeration schedule, and DFA collectively indicated that the two-cluster solution offered the most suitable fit. This is consistent with findings from prior research, which enables direct comparisons of results. Consequently, the two-cluster solution was selected for its ability to effectively differentiate clusters and form meaningful groupings in relation to neural activation during emotion down-regulation.


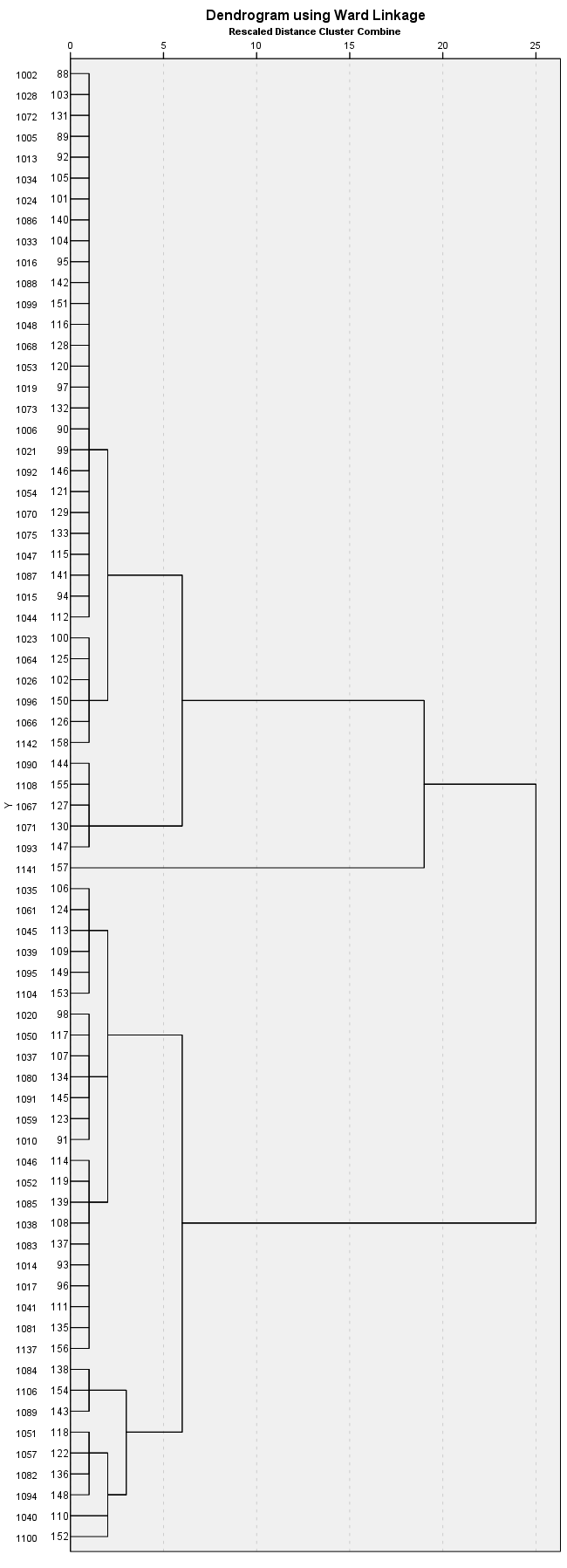


**Figure S2.** Dendrogram for the hierarchical clustering with Ward’s linkage to the determine the optimal number of clusters.

**Figure S3.** Scree plot of coefficients by stage

**Table S2.** Comparison between the entire sample of unaffected relatives and healthy controls

|  | Unaffected relatives | Healthy controls | p-value |
| --- | --- | --- | --- |
|  | M (SD) | M (SD) |  |
| *n* | 71 | 66 |  |
| **Demographic and clinical variables** |  |  |  |
| Age | 27.58 (7.02) | 29.59 (9.55) | 0.55 |
| Sex, n (% female) | 37 (52%) | 41 (62%) | 0.24 |
| Years of education | 15.15 (2.77) | 15.71 (2.26) | 0.20 |
| HDRS | 1.42 (2.11) | 1.00 (1.16) | 0.69 |
| YMRS | 0.80 (1.54) | 0.76 (1.72) | 0.54 |
| IAPS emotion regulation success* | 0.08 (0.14) | 0.14 (0.16) | **0.02** |
| FAST total | 3.70 (6.44) | 1.15 (1.67) | **.002** |
| FAST interpersonal relationships | 0.64 (1.47) | 0.26 (0.56) | 0.13 |
| Quality of life, EQ-5D index | 0.96 (0.07) | 0.97 (0.06) | 0.38 |
| Childhood trauma, total score | 31.67 (6.63) | 28.31 (4.08) | **<.001** |
| Childhood trauma, physical abuse | 5.22 (1.14) | 5.08 (0.51) | 0.28 |
| Childhood trauma, emotional abuse | 6.42 (2.08) | 5.66 (1.29) | **0.02** |
| Childhood trauma, sexual abuse | 5.19 (1.08) | 5.09 (0.52) | 0.96 |
| Childhood trauma, emotional neglect | 7.97 (3.03) | 6.59 (2.37) | .**007** |
| Childhood trauma, physical neglect | 6.87 (2.74) | 5.98 (1.72) | 0.09 |
| **Mean % signal change** |  |  |  |
| Left amygdala | 0.03 (0.28) | 0.04 (0.17) | 0.78 |
| Right amygdala | 0.05 (0.28) | 0.06 (0.20) | 0.75 |
| Left inferior frontal gyrus/VLPFC (BA47) | 0.09 (0.56) | 0.18 (0.25) | 0.24 |
| Right inferior frontal gyrus/VLPFC (BA47) | 0.16 (0.38) | 0.13 (0.33) | 0.63 |
| Left middle frontal gyrus/DLPFC (BA6) | 0.13 (0.34) | 0.17 (0.25) | 0.40 |
| Right middle frontal gyrus/DLPFC (BA8) | 0.08 (0.25) | 0.07 (0.22) | 0.85 |
| Right superior frontal gyrus/DLPFC (BA9) | 0.04 (0.33) | 0.02 (0.27) | 0.71 |
| Left middle frontal gyrus/DMPFC (BA10) | 0.02 (0.25) | 0.01 (0.21) | 0.81 |
| Left superior frontal gyrus/DMPFC (BA6) | 0.11 (0.29) | 0.11 (0.23) | 0.93 |
| Posterior cingulate gyrus (BA23) | -0.01 (0.25) | -0.05 (0.20) | 0.38 |
| Left angular gyrus (BA39) | 0.17 (0.36) | 0.21 (0.19) | 0.39 |
| Right angular gyrus (BA40) | 0.11 (0.33) | 0.05 (0.29) | 0.31 |
| Left middle temporal gyrus (BA21) | 0.07 (0.32) | 0.12 (0.18) | 0.26 |

Abbreviations: M=mean; SD= standard deviation; HDRS=Hamilton Depression Rating Scale; YMRS=Young Mania Rating Scale; IAPS=International Affective Picture System; FAST=Functioning Assessment Short Test; EQ-5D=European Quality of life – 5 Dimensions; MPFC=medial prefrontal cortex; VLPFC=ventrolateral prefrontal cortex; DLPFC=dorsolateral prefrontal cortex; DMPFC=dorsomedial prefrontal cortex. *Emotion regulation success was calculated by subtracting mean of the ratings of unpleasantness in six ‘decrease negative’ conditions from the mean of the rating of unpleasantness in the six ‘passive view negative’ conditions and values arcsine transformed. Bold text in the table indicates significant values (p<.05).

**Table S3.** Extracted mean percent BOLD signal change during emotion reactivity (passive view negative’ > ‘passive view neutral’ contrast) for unaffected relatives of patients with bipolar disorder in the two emotion regulation neural subgroups and healthy controls (HC).

|  | **Means (SD)** | | | **F** | **Three-way comparisons, p-value** | **Pairwise comparisons, p-value** | | |
| --- | --- | --- | --- | --- | --- | --- | --- | --- |
|  | **Subgroup 1 (N=39)** | **Subgroup 2 (N=32)** | **HC (N=66)** |  |  | **SG 1 vs HC** | **SG 2 vs HC** | **SG 1 vs SG 2** |
|  |  |  |  |  |  |  |  |  |
| Left amygdala | 0.23 (0.25) | 0.08 (0.25) | 0.17 (0.22) | 3.57 | **0.03** | 0.53 | 0.21 | **0.03** |
| Right amygdala | 0.26 (0.27) | 0.03 (0.31) | 0.14 (0.25) | 6.24 | **0.003** | 0.12 | 0.15 | **0.002** |
| Left inferior frontal gyrus/VLPFC | 0.19 (0.30) | -0.011 (0.45) | 0.09 (0.40) | 2.30 | 0.10 |  |  |  |
| Right inferior frontal gyrus/VLPFC | 0.09 (0.29) | -0.15 (0.41) | 0.03 (0.41) | 3.96 | **0.02** | 0.78 | 0.08 | **0.02** |
| Left middle frontal gyrus/DLPFC | 0.12 (0.22) | -0.04 (0.34) | 0.02 (0.24) | 3.67 | **0.03** | 0.14 | 0.65 | **0.03** |
| Right middle frontal gyrus/DLPFC | 0.05 (0.31) | -0.17 (0.25) | -0.05 (0.21) | 6.38 | **0.00** | 0.16 | 0.09 | **0.002** |
| Right superior frontal gyrus/DLPFC | 0.08 (0.28) | -0.12 (0.34) | -0.03 (0.28) | 4.01 | **0.02** | 0.23 | 0.37 | **0.02** |
| Left superior frontal gyrus/DMPFC | 0.18 (0.24) | 0.03 (0.34) | 0.08 (0.28) | 2.59 | 0.08 |  |  |  |
| Left middle frontal gyrus/DMPFC | 0.07 (0.19) | -0.10 (0.25) | -0.03 (0.25) | 4.73 | **0.01** | 0.15 | 0.33 | **0.01** |
| Cingulate gyrus | 0.15 (0.23) | -0.11 (0.27) | 0.02 (0.24) | 10.06 | **<.001** | **0.03** | **0.04** | **<.001** |
| Left angular gyrus | 0.16 (0.26) | -0.02 (0.23) | 0.06 (0.23) | 5.45 | **0.01** | 0.13 | 0.23 | **0.004** |
| Right angular gyrus | 0.03 (0.33) | -0.25 (0.53) | -0.10 (0.29) | 4.86 | **0.01** | 0.26 | 0.18 | **0.01** |
| Left middle temporal gyrus | 0.05 (0.21) | -0.10 (0.23) | -0.06 (0.19) | 5.75 | **0.004** | **0.02** | 0.69 | **0.01** |
| IAPS Emotion Reactivity | 1.79 (0.82) | 1.67 (1.21) | 2.11 (1.04) | 2.39 | 0.10 |  |  |  |
| Abbreviations: SD=standard deviation; SG 1=subgroup 1; SG 2=subgroup 2; HC=healthy controls; IAPS=International Affective Picture System. Bold text in the table indicates significant values (*p* < .05). | | | | | | | |  |

**
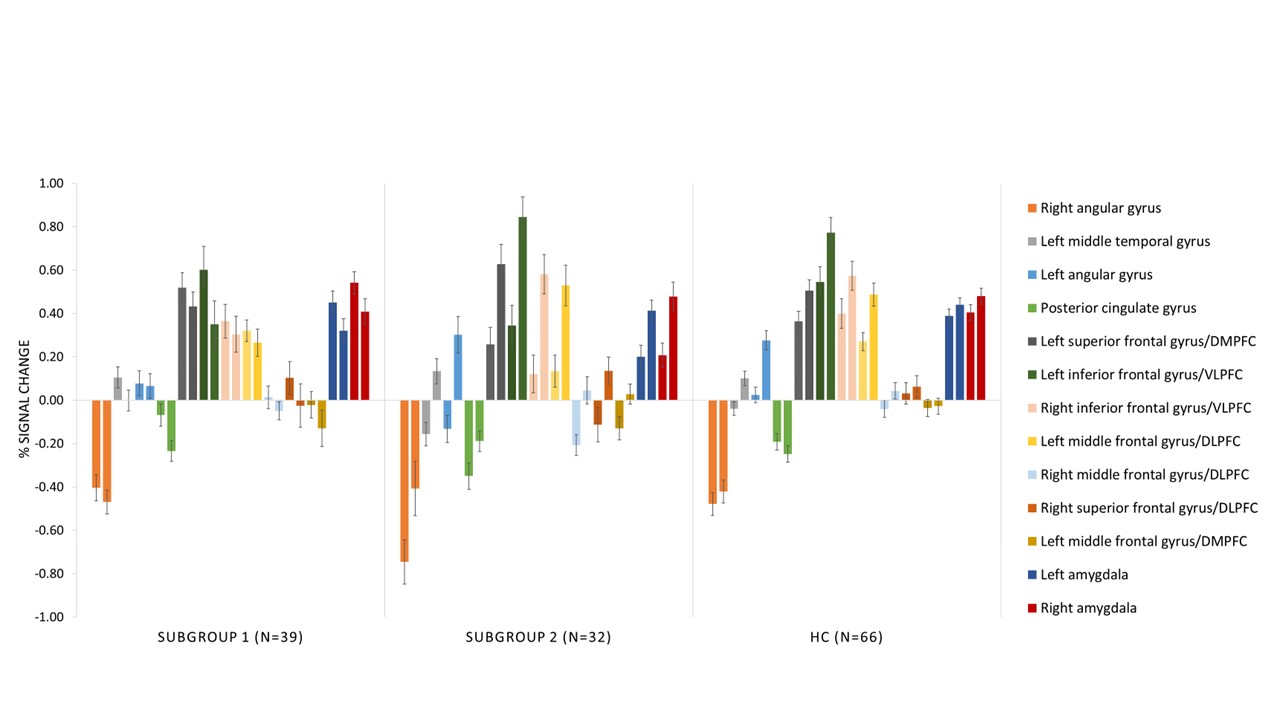
**

**Figure S4.** Mean percent BOLD signal change within the emotion regulation network during ‘passive view negative’ conditions (bars of the same color on the left) and ‘decrease negative’ conditions (bars of the same color on the right) in the two neural subgroups of unaffected relatives and healthy controls. In subgroup 1, neural activity tends to be increased during passive viewing conditions compared to conditions requiring active down-regulation of emotion, whereas UR in subgroup 2 and HC show the opposite pattern with increased neural activity during active emotion regulation conditions compared to passive viewing conditions. Error bars represent standard error of the mean.

**
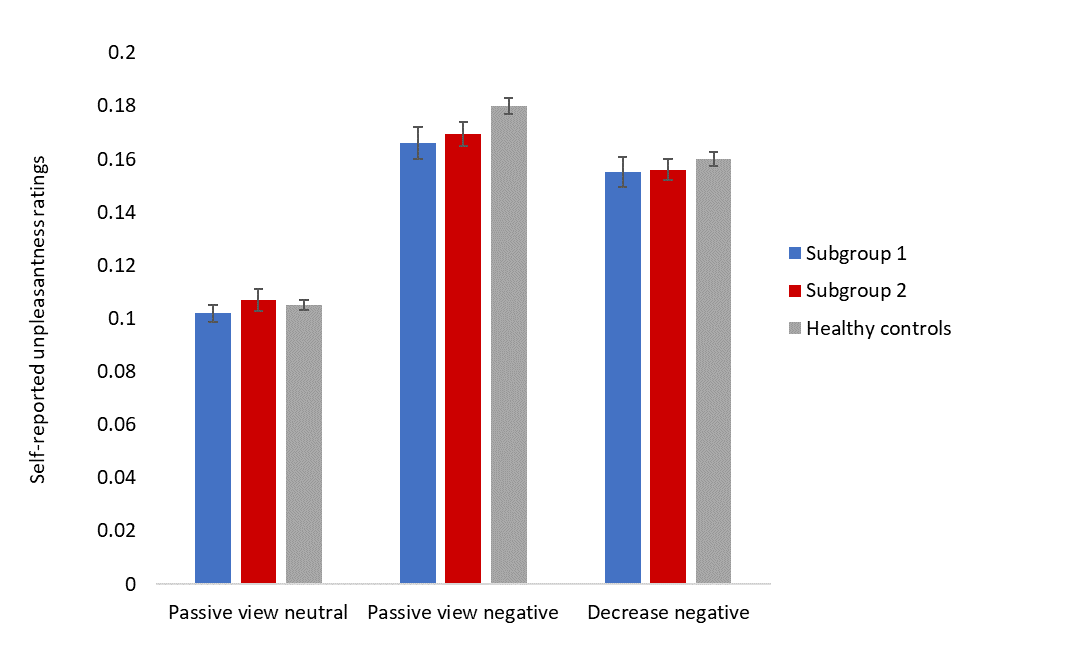
**

**Figure S5.** Behavioral in-scanner ratings of unpleasantness during the three conditions: ‘passive view neutral’, ‘passive view negative’, and ‘decrease negative’ conditions in the two neuronal subgroups of unaffected relatives and healthy controls. Values are arcsine transformed and error bars reflect standard error of the mean.

**Table S4.** Exploratory Pearson’s correlation analyses within each subgroup showing lack of significant associations between the averaged BOLD signal in the emotion regulation network and the variables that were statistically significantly different from the other subgroups.

| **CLUSTER 1** |  | CTQ Total | Behavioral success in down-regulating emotions | FERT positive faces |  |  |
| --- | --- | --- | --- | --- | --- | --- |
| Amygdalae | *r* | 0.174 | -0.079 | 0.155 |  |  |
|  | Sig. | 0.304 | 0.641 | 0.491 |  |  |
| PFC | *r* | 0.025 | 0.033 | 0.053 |  |  |
|  | Sig. | 0.885 | 0.848 | 0.817 |  |  |
| Temporo-parietal | *r* | 0.003 | 0.126 | 0.248 |  |  |
|  | Sig. | 0.987 | 0.456 | 0.266 |  |  |
|  |  |  |  |  |  |  |
| **CLUSTER 2** |  | Anxiety (HDRS items) | FAST Total | FAST Interpersonal relationships | Dot probe attentional vigilance unmasked fearful faces | FERT positive faces |
| Amygdalae | *r* | -0.075 | -0.203 | -0.058 | -0.037 | -0.063 |
|  | Sig. | 0.687 | 0.265 | 0.752 | 0.839 | 0.805 |
| PFC | *r* | -0.047 | 0.063 | 0.127 | -0.090 | -0.254 |
|  | Sig. | 0.801 | 0.732 | 0.488 | 0.624 | 0.310 |
| Temporo-parietal | *r* | -0.229 | -0.219 | -0.139 | -0.188 | -0.296 |
|  | Sig. | 0.215 | 0.229 | 0.447 | 0.303 | 0.233 |

**Table S5.** Pearson’s correlation analyses between the extracted BOLD signal change in the emotion regulation network across all participants

|  | | Left superior frontal gyrus (BA 6) | Left inferior frontal gyrus (BA 47) | Right inferior frontal gyrus (BA 47) | Left angular gyrus (BA 39) | Left middle frontal gyrus (BA 6) | Right angular gyrus (BA 40) | Right middle frontal gyrus (BA 8) | Left middle temporal gyrus (BA 21) | Right superior frontal gyrus (BA 9) | Posterior cingulate gyrus | Left middle frontal gyrus (BA 10) | Left amygdala | Right amygdala |
| --- | --- | --- | --- | --- | --- | --- | --- | --- | --- | --- | --- | --- | --- | --- |
| Left superior frontal gyrus (BA 6) | r | 1 | .813 | .671 | .711 | .794 | .494 | .562 | .610 | .584 | .532 | .670 | .360 | .415 |
|  | Sig. |  | 0.000 | 0.000 | 0.000 | 0.000 | 0.000 | 0.000 | 0.000 | 0.000 | 0.000 | 0.000 | 0.000 | 0.000 |
| Left inferior frontal gyrus (BA 47) | r | .813 | 1 | .798 | .818 | .835 | .459 | .542 | .801 | .537 | .529 | .703 | .487 | .476 |
|  | Sig. | 0.000 |  | 0.000 | 0.000 | 0.000 | 0.000 | 0.000 | 0.000 | 0.000 | 0.000 | 0.000 | 0.000 | 0.000 |
| Right inferior frontal gyrus (BA 47) | r | .671 | .798 | 1 | .621 | .657 | .550 | .592 | .588 | .623 | .584 | .638 | .499 | .547 |
|  | Sig. | 0.000 | 0.000 |  | 0.000 | 0.000 | 0.000 | 0.000 | 0.000 | 0.000 | 0.000 | 0.000 | 0.000 | 0.000 |
| Left angular gyrus (BA 39) | r | .711 | .818 | .621 | 1 | .759 | .450 | .496 | .733 | .456 | .497 | .577 | .427 | .442 |
|  | Sig. | 0.000 | 0.000 | 0.000 |  | 0.000 | 0.000 | 0.000 | 0.000 | 0.000 | 0.000 | 0.000 | 0.000 | 0.000 |
| Left middle frontal gyrus (BA 6) | r | .794 | .835 | .657 | .759 | 1 | .519 | .676 | .745 | .539 | .525 | .711 | .397 | .357 |
|  | Sig. | 0.000 | 0.000 | 0.000 | 0.000 |  | 0.000 | 0.000 | 0.000 | 0.000 | 0.000 | 0.000 | 0.000 | 0.000 |
| Right angular gyrus (BA 40) | r | .494 | .459 | .550 | .450 | .519 | 1 | .694 | .452 | .669 | .601 | .569 | .279 | .381 |
|  | Sig. | 0.000 | 0.000 | 0.000 | 0.000 | 0.000 |  | 0.000 | 0.000 | 0.000 | 0.000 | 0.000 | 0.001 | 0.000 |
| Right middle frontal gyrus (BA 8) | r | .562 | .542 | .592 | .496 | .676 | .694 | 1 | .453 | .676 | .636 | .670 | .229 | .337 |
|  | Sig. | 0.000 | 0.000 | 0.000 | 0.000 | 0.000 | 0.000 |  | 0.000 | 0.000 | 0.000 | 0.000 | 0.007 | 0.000 |
| Left middle temporal gyrus (BA 21) | r | .610 | .801 | .588 | .733 | .745 | .452 | .453 | 1 | .415 | .487 | .614 | .451 | .359 |
|  | Sig. | 0.000 | 0.000 | 0.000 | 0.000 | 0.000 | 0.000 | 0.000 |  | 0.000 | 0.000 | 0.000 | 0.000 | 0.000 |
| Right superior frontal gyrus (BA 9) | r | .584 | .537 | .623 | .456 | .539 | .669 | .676 | .415 | 1 | .654 | .769 | .234 | .348 |
|  | Sig. | 0.000 | 0.000 | 0.000 | 0.000 | 0.000 | 0.000 | 0.000 | 0.000 |  | 0.000 | 0.000 | 0.006 | 0.000 |
| Posterior cingulate gyrus | r | .532 | .529 | .584 | .497 | .525 | .601 | .636 | .487 | .654 | 1 | .666 | .505 | .502 |
|  | Sig. | 0.000 | 0.000 | 0.000 | 0.000 | 0.000 | 0.000 | 0.000 | 0.000 | 0.000 |  | 0.000 | 0.000 | 0.000 |
| Left middle frontal gyrus (BA 10) | r | .670 | .703 | .638 | .577 | .711 | .569 | .670 | .614 | .769 | .666 | 1 | .345 | .344 |
|  | Sig. | 0.000 | 0.000 | 0.000 | 0.000 | 0.000 | 0.000 | 0.000 | 0.000 | 0.000 | 0.000 |  | 0.000 | 0.000 |
| Left amygdala | r | .360 | .487 | .499 | .427 | .397 | .279 | .229 | .451 | .234 | .505 | .345 | 1 | .802 |
|  | Sig. | 0.000 | 0.000 | 0.000 | 0.000 | 0.000 | 0.001 | 0.007 | 0.000 | 0.006 | 0.000 | 0.000 |  | 0.000 |
| Right amygdala | r | .415 | .476 | .547 | .442 | .357 | .381 | .337 | .359 | .348 | .502 | .344 | .802 | 1 |
|  | Sig. | 0.000 | 0.000 | 0.000 | 0.000 | 0.000 | 0.000 | 0.000 | 0.000 | 0.000 | 0.000 | 0.000 | 0.000 |  |

**C. Emotion regulation strategies**

*Emotion regulation strategies*

For 49 UR (26 UR in subgroup 1; 23 UR in subgroup 2) and 50 HC, information was available on which strategy they reported to have used during the emotion regulation task in the fMRI scanner. The strategy mentioned most often by UR was *Cognitive reappraisal: reinterpretation*. No significant differences were found between the different subgroups. However, both subgroups of UR did make significantly less use of the strategy *Cognitive reappraisal: distancing* compared to HC (*p* < .001).

**Table S6.** Emotion regulation strategies definitions

| **Strategy** | **Definition** |
| --- | --- |
| Cognitive reappraisal: reinterpreting | Reinterpreting the elements of the situation or stimulus of the pictures to facilitate a less negative emotional response |
| Cognitive reappraisal: distancing | Adopt a new perspective, for example by changing one's personal connection to the events in the picture, or change one's psychological distance from the stimulus that elicits emotion |
| Cognitive distraction | Think about something that is unrelated to the emotion or the stimulus in order to distract oneself |
| Attentional distraction | Attentional deployment by directing one’s attention elsewhere in the picture |
| Acceptance | Thinking that the emotion or emotional stimulus is normal and should be accepted |
| Mental imagery | Modify the visual properties of the images by using mental imagery to facilitate a less negative emotional response |

**Table S7.** Frequencies of the used emotion regulation strategies used by the participants during the fMRI task. Information on the used strategies was known of 49 unaffected relatives (UR) (26 UR in subgroup 1; 23 UR in subgroup 2) and 50 healthy controls.

|  | **Means** | | | **Three-way comparisons, p-value** | **Pairwise comparisons, p-value** | | |
| --- | --- | --- | --- | --- | --- | --- | --- |
|  | **Subgroup 1** | **Subgroup 2** | **HC** |  | **SG1 vs HC** | **SG2 vs HC** | **SG1 vs SG2** |
| Cognitive reappraisal: reinterpreting | 23 | 27 | 38 | 0.135 |  |  |  |
| Cognitive reappraisal: distancing | 9 | 6 | 51 | **<.001** | **<.001** | **<.001** | 0.523 |
| Cognitive distraction | 6 | 4 | 4 | 0.256 |  |  |  |
| Attentional distraction | 1 | 2 | 8 | 0.422 |  |  |  |
| Acceptance | 5 | 8 | 8 | 0.111 |  |  |  |
| Mental imagery | 0 | 0 | 0 | 1.00 |  |  |  |
| Abbreviations: SD=standard deviation. Bold text in the table indicates significant values (*p* < .05). | | | | | | | |

**References**

Kjærstad, H. L., Damgaard, V., Knudsen, G. M., Vinberg, M., Kessing, L. V., Macoveanu, J., & Miskowiak, K. W. (2022). Neural underpinnings of emotion regulation subgroups in remitted patients with recently diagnosed bipolar disorder. *European Neuropsychopharmacology*, *60*, 7–18. https://doi.org/10.1016/j.euroneuro.2022.04.010

Morawetz, C., Bode, S., Baudewig, J., & Heekeren, H. R. (2017). Effective amygdala-prefrontal connectivity predicts individual differences in successful emotion regulation. *Social Cognitive and Affective Neuroscience*, *12*(4), 569–585. https://doi.org/10.1093/scan/nsw169

Njau, S., Townsend, J., Wade, B., Hellemann, G., Bookheimer, S., Narr, K., & Brooks, J. O. (2020). Neural Subtypes of Euthymic Bipolar I Disorder Characterized by Emotion Regulation Circuitry. *Biological Psychiatry: Cognitive Neuroscience and Neuroimaging*, *5*(6), 591–600. https://doi.org/10.1016/j.bpsc.2020.02.011

Yim, O., & Ramdeen, K. T. (2015). Hierarchical Cluster Analysis: Comparison of Three Linkage Measures and Application to Psychological Data. *The Quantitative Methods for Psychology*, *11*(1), 8–21. https://doi.org/10.20982/tqmp.11.1.p008
